# Correction: F-Formation Detection: Individuating Free-Standing Conversational Groups in Images

**DOI:** 10.1371/journal.pone.0139160

**Published:** 2015-09-22

**Authors:** Francesco Setti, Chris Russell, Chiara Bassetti, Marco Cristani

There is an error in the first sentence of the Evaluation Metrics subsection of the Experiments section. The correct sentence is: As accuracy measures, we adopt the metrics proposed in [3] and extended in [12]: we consider a group as correctly estimated if at least ⌈(T ⋅ ∣G∣)⌉ of their members are found by the grouping method and correctly detected by the tracker, and if no more than (1−T) ⋅ ∣G∣ false subjects (of the detected tracks) are identified, where ∣G∣ is the cardinality of the labelled group G, and T Є [0,1] is an arbitrary threshold, called tolerance threshold.
